# The Effect of Five-Day Dry Immersion on the Nervous and Metabolic Mechanisms of the Circulatory System

**DOI:** 10.3389/fphys.2020.00692

**Published:** 2020-07-10

**Authors:** Vasily B. Rusanov, Ludmila Kh. Pastushkova, Irina M. Larina, Anna G. Chernikova, Anna G. Goncharova, Andrei M. Nosovsky, Daria N. Kashirina, Alexander G. Brzhozovsky, Nastassia Navasiolava, Alexey S. Kononikhin, Anna R. Kussmaul, Marc-Antoine Custaud, Evgeny N. Nikolaev

**Affiliations:** ^1^Institute of Biomedical Problems of the Russian Academy of Sciences, Moscow, Russia; ^2^Skolkovo Institute of Science and Technology, Skolkovo, Russia; ^3^University of Angers, Angers, France; ^4^V.L. Talrose Institute for Energy Problems of Chemical Physics, N.N. Semenov Federal Center of Chemical Physics, Russian Academy of Sciences, Moscow, Russia

**Keywords:** circulatory system, regulatory mechanisms, proteomics, dry immersion, effects of microgravity

## Abstract

The purpose of the study was to investigate the regulatory and metabolic changes in the circulatory system when simulating microgravity conditions in a five-day dry immersion. These changes reflect the adaptation processes characteristic for the initial stages of a space flight or a short-duration space flight. Studies were conducted with 13 healthy male volunteers aged 21 to 29 years. The assessment of regulatory and metabolic processes in the circulatory system was based on the heart rate variability (HRV) and urine proteomic profile analysis. It was found that the restructuring of hemodynamics during 5 days hypogravity begins with the inclusion of the nervous circuit of regulation, and for manifestations at the body fluids protein composition level and activation of the metabolic regulation, these periods are apparently insufficient. Perhaps this is due to the fact that the metabolic regulation, being evolutionarily ancient and genetically determined, is more stable and requires more time for its pronounced activation when stimulated by extreme life conditions.

## Introduction

It is well-known that during space flight, the effect of microgravity has a direct influence on the cardiovascular system, including bioelectric changes in the myocardium and its remodeling, autonomic reflexes and associated physiological processes (Lees, 2005; Shen and Frishman, 2019). Moreover, a normal level of functioning is maintained due to changes in regulatory adaptive mechanisms (Otsuka et al., 2018). The heart rate (HR) reflects cardiovascular homeostasis and is associated with various regulatory influences. During space flight, the resting HR varies slightly compared to terrestrial conditions, and the main changes are manifested in autonomic balance shifts (Baevsky, 1997). From this point of view, we consider the stable level of functioning (resting HR, for instance) as a homeostatic level of regulation, and observed changes in the HR autonomic regulation are considered as adaptive.

For developing the preventive measures aimed at eliminating the negative effects of microgravity and maintaining the cosmonauts operability, studies in model experiments are used, the relevance of the tasks in which increases owing to the planned Moon and Mars expeditions outside the low Earth.

“Dry” immersion (DI; reproduction of the supportlessness conditions) is a model used in gravitational physiology to simulate the effects of microgravity on body systems. DI reproduces cardiovascular, motor and other changes similar to those observed in space flights. Moreover, the severity and directionality of adaptation processes under immersion exposure are with those observed in space flights of various durations (Tomilovskaya et al., 2019).

The purpose of the study was to investigate the regulatory and metabolic changes in the circulatory system when simulating microgravity conditions in a five-day DI as a reflection of the adaptation processes in this system characteristic for the initial stages of space flight or for space flight of short duration.

## Materials and Methods

The studies were conducted at the “dry immersion” stand of the Institute of Biomedical Problems of the Russian Academy of Sciences (IBMP RAS), with the participation of 13 healthy male volunteers aged 21 to 29 years. All volunteers were allowed to participate in the study by a medical expert commission. The research procedures and methods were reviewed and approved by the IBMP RAS Commission on Biomedical Ethics (protocol No. 273 dated June 23, 2010), and the written voluntary Informed consent was obtained from the participating in the study testers. To simulate the physiological effects of microgravity, the subjects were immersed in water in lying down to the level of the shoulder upper third (water *t* = 33–34°C) without contact with it, since they were separated from the water by a waterproof, freely fixed to the sides fabric, being freely “hung out” in an immersion environment (Tomilovskaya et al., 2019). During DI, the volunteers were not subjected to either pharmacological or any other additional influences aimed at preventing developing adaptive shifts in physiological systems.

Regulatory and metabolic processes in the circulatory system were evaluated on the basis of heart rate variability (HRV) and urine proteomic profile analysis, since HRV indicators reflect the general state of regulatory mechanisms, and the molecular level of heart rate physiological regulation is reflected in the extracellular fluid protein composition studied by proteomics based on mass spectrometry (Pastushkova et al., 2019).

We estimated the total, for 5 days, impact of immersion effects on these processes. Therefore, the data obtained only before and after DI (at 2 days before the start of the experiment and at + 1 day after the end of the exposure) have been analyzed.

The HRV analysis was carried out in 5-min ECG samples at rest in supine position. The cardiovascular regulatory mechanisms condition was assessed according to the recommendations developed by the European cardiological and North American electrophysiological Societies (No Author List, 1996). The following indicators were analyzed:

Heart rate (bpm) – heart rate, reflects the current level of functioning of the circulatory system.

pNN50 (%) – is the numbe of pairs of adjacent intervals differing by more than 50 ms, in% of the total number of cardiointervals in the array, an indicator of the parasympathetic regulatory branch prevalence over the sympathetic.

Stress index (conventional units) – stress index, index of regulatory systems stress, characterizes the degree to which the activity of sympathetic regulation mechanisms prevails over parasympathetic ones. The SI is used in occupational and environmental studies to quantify the physical and mental stress of work processes (Quendler et al., 2017), to assess the HRV seasonal features (Markov et al., 2016), to evaluate the activation of the adaptive capabilities of the organism, expressed through an imbalance of the autonomic nervous system due to sympathetic predominance (Konstantinova et al., 2017). The SI is included in calculated HRV parameters in the Kubios HRV software^1^.

The stress-index (SI) is calculated by geometric methods for assessing the RR intervals distribution over the period of investigation. For the purpose, variation curve (histogram of RR intervals distribution) is built and main characteristics are determined including Mo (mode), AMo (mode amplitude), and MxDMn (variation range).

Mode is the most common interval value in a dynamic series. In case of normal distribution and high process stationarity, Mo differs little from mathematical expectation (M).

AMo (mode amplitude) is a number of intervals corresponding to mode value in% to sample size.

Variation range MxDMn shows the degree of interval variativity in a current dynamic series. It is calculated from the difference of maximum (Mx) and minimum (Mn) intervals and, therefore, can be distorted by arrhythmias or artifacts.

The variation pulsometry data is used to calculate the stress-index.

SI = AMo/(2Mo × MxDMn). The SI characterizes the activity of sympathetic regulation. Activation of the sympathetic regulation during mental or physical stresses manifests itself by rhythm stabilization, decrease of the range of interval duration, and increase of the number of intervals with similar duration (AMo growth). Normal SI fluctuates within 80–150 conventional units. It is very sensitive to the sympathetic tone rise.

The SI is known in Russia since 1970-th and it is almost similar to triangular index, total number of all NN intervals divided by the height of the histogram of all NN intervals measured on a discrete scale with bins of 7⋅8125 ms (1/128 s).

Power HF (mc^2^) – is the raw power of HRV high-frequency component from the total power (sum of HF, LF, and VLF spectral components), the relative level of parasympathetic activity.

Power LF (mc^2^) – is the raw power of HRV low-frequency component from the total power (sum of HF, LF, and VLF spectral components), the relative activity of the subcortical sympathetic vasomotor center in the medulla oblongata.

LF and HF may also be measured in normalized units (n.u.) which represent the relative value of each power component in proportion to the total power minus the VLF component.

LF/HF (conventional units) is the ratio of the high-frequency and low-frequency components spectral power, an indicator characterizing the balance of sympathetic and parasympathetic influences and the relative activity of the subcortical sympathetic center.

Changes in the extracellular fluid protein composition were evaluated in the study of urine proteome. Urine collection was carried out in the daytime, in the form of a freely separated 2-nd morning fraction, which was subsequently prepared for mass spectrometric analysis, according to the standard protocol (Nkuipou-Kenfack et al., 2014). Urine samples were subjected to preparation, consisting of stages: recovery, alkylation, protein deposition, and proteolysis using trypsin.

The shotgun proteomics methodology was used for semi-quantitative analysis of the obtained polypeptide mixture. The mixture was separated by liquid chromatography (Agilent 1100, Agilent Technologies Inc., Santa Clara, United States) in three repetitions and analyzed by 7T LTQ-FT Ultra hybrid mass spectrometer (Thermo, Bremen, Germany) of ion cyclotron resonance combined with a linear quadrupole ion trap. A column with reversed phase ReproSil-Pur C18 (particle diameter 3 μm, pore diameter 100 Å, Dr. Maisch GmbH, Ammerbuch-Entringen, Germany) manufactured using a capillary emitter (Pico-tip, New Objective Inc., United States), has been used for chromatography.

The peptides mixture was analyzed using the Xcalibur software (Thermo, Bremen, Germany) in data dependent aqusition (DDA) mode. Proteins were identified with the help of MaxQuant software, using the SwissProt database. Only proteins that were identified by at least 2 peptides with one which is unique to the protein were subjected to further analysis.

Two hundred fifty six different proteins were identified after chromatography-mass spectrometric analysis of all urine samples by UniProtKB nomenclature.

The method of principal components was used for statistical analysis (Nosovsky et al., 2018), and the Perseus software package was used to determine molecular functions and biological processes with the participation of identified proteins.

## Results

When assessing the dynamics of the mean group values of HRV indicators characterizing the modulating effect of the ANS sympathetic (SI) and ANS parasympathetic (pNN50) branch after DI exposure, a shift in the autonomic balance toward sympathetic activation was revealed. However, other indicators characterizing the certain aspects of the regulatory mechanism have not changed. High-frequency oscillations (HF) and low-frequency oscillations (LF), also had unreliable changes after immersion (Table 1).

**TABLE 1:** Heart rate variability indices before and after 5-days dry immersion.

Indicator	Before immersion (M)	After immersion (M)
pNN50 (%)	23,97	15,19*
SI (c.u.)	117,03	183,07*
TP(mc^2^)	4623,83	3430,76*
HF(mc^2^)	1611,53	1123,84
LF(mc2)	2912,3	2306,92
HF(n.u.)	36,2	32,1
LF(n.u.)	63,4	67,9
LF/HF(c.u.)	3,60	3,92
HR(bpm)	76,30	84,92

* Significant changes after immersion (the Wilcoxon signed-rank test *p* < 0.05).

The activation of sympathetic nerve regulatory mechanisms occurred as a result of the interaction of homeostatic and adaptive mechanisms in order to maintain cardiovascular homeostasis and increase adaptive capabilities and mobilize functional reserves. Apparently, after 5-day immersion, the mechanisms of the nervous regulation of the heart rhythm are turned on primarily. At the same time, systemic homeostasis does not change, as evidenced by the stability of heart rate parameter, an integral physiological indicator of most stress-limiting body systems, including the cardiovascular system.

One of the regulation systems is the humoral-metabolic system, which exerts an effect through the activity of biologically active substances circulating in body fluids and tissues, including of protein nature (proteins). From the general list of 256 proteins, detected prior to the immersion exposure in the urine proteomic profile, we identified 6 proteins that reflect regulatory processes in the circulatory system: CADM4, Immunoglobulin heavy alpha-1 (IGHA1), TF, AXL, Gal-3BP, and MXRA8.

The physiological (and regulatory) role of the mentioned proteins was clarified by manual annotation using open databases.

**FIGURE 1:**
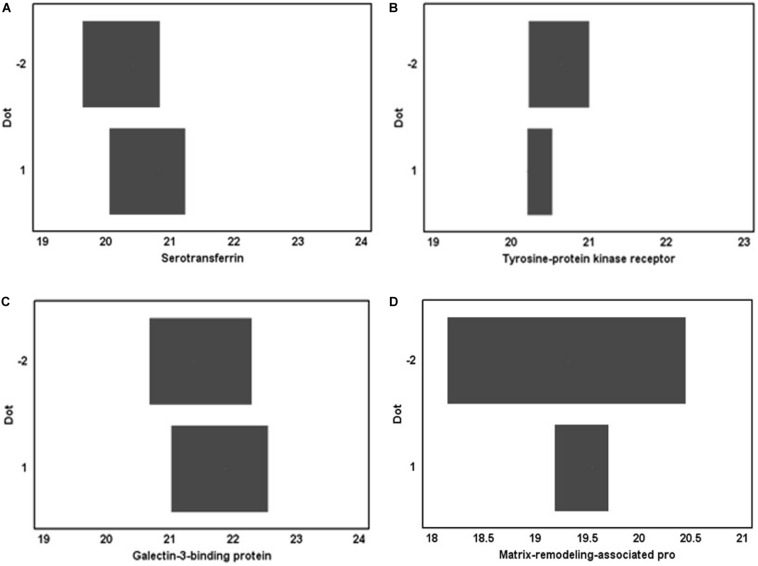
Proteins identified in the urine proteomic profile before DI and their concentration after immersion exposure. **(A)** serotransferrin, **(B)** tyrosine-protein kinase receptor, **(C)** galectin-3-binding protein, and **(D)** matrix-remodeling-associated protein.

However, statistical analysis showed the absence of significant changes in the concentrations of these proteins in the urine proteome after 5-day immersion (Figure 1). Perhaps this is due to the fact that the metabolic contour of regulation, is more stable and requires more time for its pronounced activation when stimulated by extreme life conditions. In this regard, it seems logical that the 5-day duration of immersion does not change the metabolic mechanisms of regulation, which are reflected in the urine proteomic profile.

## Discussion

Our analysis before exposure to a five-day DI revealed the presence of proteins associated with the regulation of cardiovascular system. In our opinion, this combination provides a balance of cardiovascular homeostasis, and is associated with complex interacting processes of atherogenesis, neoangiogenesis, calcium channels activation, changes in cell adhesion, and transmembrane properties, and extracellular matrix metabolism. At the tissue level, signaling proteins are involved in changing the vascular wall stiffness and the endothelium properties, affecting the peripheral vascular resistance.

Together with HRV indices, proteome signaling molecules reflect the nervous and metabolic control mechanisms condition and realize a homeostatic role, and the orientation and intensity of changes in both patterns determine the adaptation strategy of the circulatory system in the acute period of body adaptation when simulating the conditions and physiological effects of microgravity.

CADM4-membrane protein is involved in the transmission of a nerve impulse as a stimulus of cardiac contraction (Zeng and Yelon, 2014). Nectins and nectin-like molecules (Necls)/Cadms are Ca2 + -dependent adhesion molecules of the immunoglobulin superfamily expressed in cell of most types. Nectins mediate not only homotypic, but also heterotypic cell adhesion, in contrast to classical cadherins, which are involved only in homophilic adhesion. The participation of nectins and Necls in the organogenesis of sensory organs – the eyes, inner ear, and cerebral cortex, and in various developmental processes, including the formation of synapses, axons, and myelination, helps to understand the connecting role of cell adhesion molecules in the implementation of nerve impulse transmission and the features of its effect (Lambert et al., 2016).

Immunoglobulin heavy alpha-1 is a membrane-bound or secreted glycoprotein produced by b-lymphocytes. Apparently, it is the main regulatory protein that affects the arteries, veins and cardiac muscle stiffness and elasticity, mediating the features of cardiac contraction in accordance with the rigidity of the vascular wall. The IGHA1 associations with the influence of main factors determining the vascular wall stiffness and elasticity and with chronic vascular diseases has been proved. The proteins associated with atherosclerosis are: IGHA1, serum amyloid A1 (SAA), and four complement cascade proteins: complement factor B (CFAB), complement C2 (CO2), complement C3 (CO3), and complement C1s (C1S) – a subcomponent that is proposed to be considered as biomarkers of the cardiovascular risk (Gordon et al., 2018). Cavassan et al. (2019) identified immunoglobulin heavy constant alpha 1 and seven more proteins that assotiate with the presence of congestive heart failure, hypertension, and the patient’s age.

TF is a plasma glycoprotein that is synthesized in the liver. It is believed that the study of serotransferrin metabolism is important in the study of the vascular diseases pathogenesis (Golizeh et al., 2017). An association of endothelial damage with a violation of the rheological properties of blood and serotransferrin was noticed (Orlov et al., 2012).

AXL is expressed mainly in the vascular endothelium, and is involved in the regulation of proliferation, migration, invasion, and the formation of endothelial cells (Li et al., 2017). It was shown that Gas6/UFO signaling plays an important role in the survival of endothelial cells during the development of acidosis (Gustafsson et al., 2009). AXL is involved in the angiogenesis regulation. It was found that patients with heart failure and severe left ventricular remodeling processes have a higher level of sAXL, which indicates the potential role of the GAS6-AXL system in the pathophysiology of these processes (Caldentey et al., 2019). Increased AXL expression in arteries was revealed in patients after coronary artery bypass grafting. AXL stimulates STAT1 signaling (a member of the transcription factors of signal converters and transcription activators family) by inhibiting SOCS1 (cytokine signaling suppressors) in activated smooth muscle cells during venous transplant remodeling (Batchu et al., 2015).

Gal-3BP – stimulates intercellular adhesion and regulates intercellular transmission of pro-inflammatory signals, including in macrophages originating from human monocytes (DeRoo et al., 2015; Gleissner et al., 2017; Sun et al., 2017). Gal-3BP levels are independently associated with markers of metabolic and inflammatory response. Subcellular microvesicles containing galectin-3-binding protein are involved in the transmission of information from cell to cell and regulate immunity, hemostasis, transferring at the cell membrane allotted areas (size from 200 to 1000 nm), usually called microparticles (MP). The number and, in particular, the MP composition seem to reflect the state of their parent cells. Therefore, the components of the MPs protein composition may have great potential as clinical biomarkers studied by proteomics. The overexpression of galectin-3-binding protein was observed in patients with venous thromboembolism and systemic autoimmune diseases. Thus, this protein can act as a marker of “pathogenic” subcellular microvesicles (Nielsen et al., 2017). It is associated with the process of atherosclerosis and its level increasing is prognostically unfavorable for patients with coronary artery disease, since this protein is highly expressed in atherosclerotic plaques and its content correlates with the clinical manifestations of ischemia. It is supposed that quantitative characteristics of this protein level help to monitor the processes of atherosclerotic plaques destabilization (Hao et al., 2019).

MXRA8 through intermediaries is associated with cell adhesion proteins, participating in the processes of normal permeability at the tissue level (Yonezawa et al., 2003; Zhang et al., 2018), and probably, through this, taking part in the regulatory processes of the circulatory system.

## Conclusion

Under the conditions of “dry immersion” there is a change in the autonomic regulatory mechanisms of the circulatory system. This is due to hypogravity conditions and is primarily associated with elimination of the support reaction, redistribution of body fluid volumes and loss of water by the body. This changes cause energy-metabolic shifts that require activation of the corresponding regulatory mechanisms (Larina et al., 2011). This is similar to the physiological effects observed in space flights. Most of these changes are characterized by both very rapid progression and recovery upon returning to normal conditions – as a result it is believed that microgravity causes functional changes in the cardiovascular system that are adaptive in their nature (Aubert et al., 2016; Garrett-Bakelman et al., 2019). In addition, Sun et al. (2015) revealed phase changes in HRV in three-day and five-day immersion dynamics, manifested in the ANS sympathetic branch modulating effects decrease in the first two days of DI.

The hemodynamics restructuring during 5-days hypogravity begins by the involvement of the “nervous contour of regulation,” and for manifestations at the level of body fluids protein composition, activation of the “metabolic contour of regulation,” this DI duration is, obviously, insufficient. The obtained data require further studies in the direction of clarifying the time at which the “metabolic regulatory contour” become active. In the framework of a unified concept about a hierarchy of control levels in biological systems, it is important to evaluate the dynamics of the human body biological fluids proteome, in particular urine, as the most accessible of them for research in relation to the control mechanisms of regulation.

## Data Availability Statement

The datasets generated for this study are available on request to the corresponding author.

## Ethics Statement

The studies involving human participants were reviewed and approved by Institute of Biomedical Problems of the Russian Academy of Sciences Commission on Biomedical Ethics. The patients/participants provided their written informed consent to participate in this study.

## Author Contributions

IL, VR, EN, and M-AC conceived and designed the experiments. LP, VR, DK, and NN performed the experiments. AC, AB, ASK, AG, NN, and AN analyzed the data. VR, LP, AC, AG, and ARK wrote the manuscript. All authors read and approved the final manuscript.

## Conflict of Interest

The authors declare that the research was conducted in the absence of any commercial or financial relationships that could be construed as a potential conflict of interest. The handling editor is currently organizing a Research Topic with one of the authors M-AC.

## Footnotes

1 https://www.kubios.com/about-hrv/
